# Symbiosexual Attraction: An Integrated Mixed-Methods Study

**DOI:** 10.1007/s10508-024-02857-x

**Published:** 2024-04-08

**Authors:** Sally W. Johnston

**Affiliations:** Human Sexuality Department, California Institute of Integral Studies, 1453 Mission Street, San Francisco, CA 94103 USA

**Keywords:** Symbiosexual, Polyamory, Nonmonogamy, Sexual attraction, Sexual orientation, Queer

## Abstract

A recent review of cultural and academic discourse presented evidence that some people experience attraction to two (or more) people in a preexisting relationship. This phenomenon, symbiosexuality, is understudied in the field of sexuality. Lack of recognition and validation for this attraction, including in the polyamorous community, may be negatively impacting those who experience symbiosexual attraction. I conducted an integrated mixed-methods analysis of secondary data from the 2023 *The Pleasure Study* to learn more about symbiosexual attraction. Findings from this study support the hypothesis that people experience symbiosexual attraction, which they describe as an attraction to the energy, multidimensionality, and power shared between people in relationships. Further, findings from this study indicate that a diverse group of people experience symbiosexual attraction and, while unanticipated, symbiosexual attraction can be a strong, frequent, and/or pervasive experience. These findings push the boundaries of the concepts of desire and sexual orientation in sexuality studies and challenge the ongoing invisibility and invalidation of and stigma and discrimination against such attractions, within both the polyamorous community and our broader mononormative culture.

## Introduction

What if a person’s primary attractions are not oriented toward individuals? A recent review of cultural and academic discourse presented evidence that some people experience attraction to preexisting relationships between two (or more) people (Johnston, 2023). This phenomenon, symbiosexuality, a term first coined and described in a poster presentation at the Society for the Scientific Study of Sexuality conference, is defined as “the individual experience of sexual and/or romantic attraction to people in relationships” (Johnston & Schoenfeld, 2021, para. 3). It is the attraction to the relationship and/or energy shared between people that makes symbiosexuality distinct from plurisexualities such as bisexuality or pansexuality which are defined as attractions to more than one gender or attractions to all genders (Hayfield, 2021). Symbiosexuality is also distinct from an interest in or preference for relationship structures involving three or more people (triads, quads, etc.), as this interest does not necessarily imply an experience of attraction to relationship dynamics between people (preexisting or not).

In my recent review, I found evidence of symbiosexuality in essays, memoirs, dating apps, discourses on the sexual identity label known as the unicorn, as well as outside Western discourses on desire and sexuality including ancient Middle Eastern texts and Indigenous narratives (Johnston, 2023). Despite compelling evidence of its existence, attraction to the relationships between people, as a phenomenon, remains largely uninvestigated. The purpose of this study was to document the existence and nature of symbiosexual attraction. Studying this multidirectional, multiobject attraction pushes the boundaries of the concepts of desire and sexual orientation in sexuality studies and challenges the ongoing invisibility and invalidation of and stigma and discrimination against such attractions, within both the polyamory community and our broader mononormative culture.

### Literature Review

Studying unrecognized sexualities is important. When we are not asked about certain experiences in our lives or are not given language for those experiences, we may not be aware of those experiences, or we may not consider them as valid or relevant (Blumer, 1969). We come to know what is possible, intelligible, and real in part through the act of describing and naming (Kean, 2018). Attention to and language for sexual experiences can facilitate personal and scientific exploration of those experiences, can expand knowledge of human sexualities, and can also inform human sexualities (Foucault, 1978; van Anders, 2015). This process has been evident in the research and recognition of asexuality as a unique identity that expands queer conceptions of sexuality (Rothblum et al., 2020). In recent years, there has been a flourishing of academic studies, social recognition, and community support for those who do not experience spontaneous and/or consistent sexual attraction (Asexual Visibility & Education Network [AVEN], 2023). Academic recognition of asexuality—as well as description and use of the term—has offered validation for an experience of sexual attraction—or more accurately lack thereof—that was previously rendered invisible or pathologized as a medical problem (Bogaert, 2004, 2015). Asexuality research has also expanded knowledge on the diversity of human experiences relevant to sexual attraction in people’s lives.

Similar pathways for validation and recognition have opened for people who experience desire for more than one gender (plurisexuals), like people who identify as bisexual or pansexual (Galupo et al., 2015; Hayfield, 2021). Galupo (2018) found that recognition, visibility, and pleasure seeking have been facilitated for people who experience plurisexual attractions by their ability to mark their desires through self-identification with available descriptive terms like bisexual. Further, people are able to strategically use different labels (bisexual, pansexual, polysexual, etc.) to describe different experiences of desire (Galupo, 2018).

Despite gains through research and activism, plurisexualities continue to battle for recognition and validation in a world that stigmatizes plurisexualities (Schrimshaw et al., 2018) and assumes and privileges monosexual desires, identities, and lifestyles (Gonzalez et al., 2017). In her studies of plurisexual invisibility and invalidation, specifically for bisexual and pansexual individuals, Hayfield (2021) outlines potential harms, including trouble with sense of self and decreased health and well-being due to lack of recognition and validation for these identities across cultural, social, and academic fields. The negative impacts of marginalization or invalidation for plurisexualities may be heightened for those with intersecting marginalized identities (gender, race/ethnicity, religion, etc.) who face discrimination in other arenas (Collins & Bilge, 2020; Morgan et al., 2018). While the impact of lack of recognition for those who experience symbiosexual attraction is unknown, it is likely that those who experience this attraction, which appears as a multiperson attraction, are at risk of negative impacts like those experienced by people with plurisexual attractions.

In recent years, researchers have debated the relevance and importance of multipartner, multidirectional sexual preferences, like polyamory, and how this may or may not be considered part of one’s sexual identity or orientation (Cardoso & Rosa, 2021; Klesse, 2014; Manley et al., 2015; Robinson, 2013; Tweedy, 2011). Instead of arguing that multipartner preferences challenge unidirectional conceptions of sexual attraction and orientation, researchers have considered whether identities such as bisexuality and nonmonogamous relationship preferences fit within established sexual orientation boundaries as multiple unidirectional experiences. The notion that attractions and orientations can only be directed toward individual people (even if those people vary by gender) obscures the possibility of symbiosexuality. Klesse (2014) addresses this directly when he talks about how dominant theories of erotic desire and sexual orientation “arrest the multi-directional flows of desire” (p. 95).

Interestingly, despite lack of recognition and validation for symbiosexual attractions, there is a term used in the polyamorous community as well as in other nonmonogamous communities specifically to describe people who are interested in sex and relationships with couples: unicorn. This term typically refers to bisexual, queer women who are willing to engage in dynamics with heterosexual couples (Johnston, 2024). While the term only implies a willingness or interest to engage in dynamics with couples and not (necessarily) an attraction to their preexisting relationship, the existence of the term both evidences symbiosexual attraction and discredits it. Evoking notions of myth and fetish, the term unicorn perpetuates invalidation of and discrimination against people who seek these dynamics whether they are motivated by symbiosexual attraction or not.

Further, in addition to the negative connotations of the term unicorn, the large and rapidly growing polyamorous community holds a belief that the power dynamics involved in sex and relationships with couples (sometimes referred to as couple’s privilege) function as inherently unethical (Johnston, 2024). The term unicorn hunting is used to highlight and discourage the pursuit of single women by heterosexual couples as well as to discourage bisexual and pansexual women’s receptivity to heterosexual couples because of the possibility of abuse of power (Johnston, 2024). The polyamory community discourages, criticizes, treats as an “out-group,” and declares emotionally unhealthy those who come with curiosity or seek advice about sex and relationships with established couples (Johnston, 2024). As a result, those who experience symbiosexual attraction face unique discrimination in the very places they may seek community, support, and information.

Further, regardless of how the term unicorn is thought about in nonmonogamous spaces, it fails to distinguish between those who are simply willing to engage in these dynamics from those who may be driven by a genuine attraction or orientation toward two (or more) people in relationship. These are two distinct phenomena. In making my case for the existence of the phenomenon of symbiosexuality (Johnston, 2023), I include a quote from essay in *Vanity Fair* from a person who self identifies as a unicorn,

I’m not sure if Aristotle was a unicorn, but the whole is definitely greater than the sum of a couple’s parts. A unicorn not only dates the individuals, but also dates the relationship. This third force to flirt with is undoubtedly the most interesting one. (Giuliani, 2021, p. 1)

This quote alludes to something beyond a willingness to engage in dynamics with two people. It implies that some people, whether they use the term unicorn or not, experience a unique attraction to a dynamic that is “greater than the sum of [its] parts”: an attraction worthy of its own label and of academic attention. In light of the power of attention and language to describe and validate human sexual experiences, the risks associated with plurisexual invisibility and invalidation, and stigma and discrimination within the polyamorous community toward those interested in couples, documentation of symbiosexuality as valid experience of attraction is warranted.

### Research Questions

I analyzed and combined qualitative and quantitative data from *The Pleasure Study* (Harvey et al., 2023) to address the following research questions about both the existence and nature symbiosexual attraction:

1. Do people experience symbiosexual attraction? If so, what are their demographic and personal characteristics?
2. How do people describe symbiosexual attraction? To what relationship dynamics are they specifically drawn?
3. How significant is symbiosexual attraction in people’s lives?

Based on my review of discourse on attraction to people in relationships (Johnston, 2023) and my study of unicorn identity in an online polyamorous community (Johnston, 2024), I hypothesized that:

#### H1

People experience symbiosexual attraction. Those who experience this attraction will represent diverse demographic profiles and personal characteristics, but because the unicorn identity is specifically associated with bisexual/pansexual women this attraction may be more prevalent in this population.

#### H2

People who experience symbiosexual attraction will describe a variety of dynamics between people in relationships to which they are specifically drawn.

#### H3

People will report a range of awareness levels, frequencies, and strength of their symbiosexual attraction; for some, this experience will be significant and/or pervasive.

## Method

I conducted an exploratory integrated mixed-methods analysis (Creswell et al., 2011; Fetters et al., 2013) of secondary data from Stage 2 of *The Pleasure Study* (Harvey et al., 2023) to examine the existence and nature of symbiosexual attraction. *The Pleasure Study* was designed to investigate the relationship between gender identity/expression and sexual pleasure. In Stage 2, researchers sought to investigate why femininity and those with marginalized gender identities are associated with increased performances of sexual pleasure, the primary finding from stage one (Harvey, 2020, 2021). Stage 2 included questions that inquired about gender, sexual orientation, relationship practices, culture, education, performance of sexual pleasure (i.e., performing/faking orgasm, performing/faking pleasure with sounds such as moaning or gestures such as back arching or muscle clenching), and specifically included questions about romantic and sexual experiences with couples.

### Participants

In Stage 2 of *The Pleasure Study* (Harvey et al., 2023), researchers specifically recruited queer (LGTBQ) and nonmonogamous populations using convenience sampling and snowball sampling (Dunne, 2002). Participants were recruited from online community spaces (such as *Facebook*, *Reddit*, *Instagram*, *Meetup*, and community listservs) using digital flyers and posts advertising the study and including a link to the survey. Participants had to be English speaking and express their consent prior to participating in the survey. Participants were excluded from the study if they were under 21 or reported that they never had sex (sex was self-defined).

Data collection for Stage 2 of *The Pleasure Study* is currently underway. As of May 1, 2023, the sample included a total of 373 survey participants and 42 interviewees (interviewees were part of the survey sample). The sample included a larger than average portion of queer sexualities (74.4%) and genders (35.7%), as well as nonmonogamous relationship identities (75.0%). Participants were predominantly between 21 and 40 years old (75.5%), White (66.6%), not religious (74.7%), attained a bachelor’s degree or higher (69.5%), middle class (50%), living in the USA (70.4%), and living in urban areas (83.8%). See Table 1 for the demographic distributions of *The Pleasure Study* participants.

**Table 1:** *Pleasure Study* survey respondents

Variable	Frequency	Valid percentage
Age (in years)		
21–30	123	40.7%
31–40	105	34.8%
41–50	53	17.5%
51–60	17	5.6%
61–70	3	1.0%
71–80	1	0.3%
Missing	71	
Race/Ethnicity^a^		
Asian/South Asian	17	5.0%
Arab	0	0.0%
Black/African American	39	11.5%
Hispanic/Latinx/Spanish	29	8.6%
Indigenous Peoples^b^	12	3.6%
Jewish	23	6.8%
Middle Eastern/North African	2	0.6%
White (Only)	225	66.6%
Missing	35	
Religion		
Important	83	25.3%
Christian/Catholic	24	
Jewish	10	
Pagan/Witchcraft	14	
Spiritual	8	
Not Important	245	74.7%
Missing	45	
Education		
Less than High School	1	0.3%
High School/GED	14	4.2%
Some College/Associates	87	26.0%
Bachelors	104	31.0%
Masters	102	30.4%
Doctoral/Professional	27	8.1%
Missing	38	
Social Class		
Working	118	35.5%
Middle	166	50.0%
Upper/Middle, Upper	48	14.5%
Missing	41	
Country/Region		
USA (41 States)	228	70.4%
Mid-West	36	
North-East	53	
South-East	56	
South-West	18	
West	51	
Outside USA (25 Countries)	96	29.6%
Australia	6	
Brazil	1	
Bulgaria	1	
Canada	54	
Czech Republic	1	
Finland	1	
France	1	
Germany	7	
Honduras	1	
India	3	
Ireland	1	
Italy	1	
Kenya	1	
Lithuania	1	
Luxembourg	1	
Mexico	1	
Nepal	1	
New Zealand	1	
Nigeria	1	
Poland	1	
Portugal	1	
Spain	1	
Ukraine	1	
UK	6	
Venezuela	1	
Community Type		
Rural	45	16.2%
Urban	280	83.8%
Missing	39	
Sexual Orientation^c^		
Asexual Only	4	1.1%
Heterosexual Only	56	15.6%
Gay, Gay/Queer Only	22	6.1%
Lesbian, Lesbian/Queer Only	14	3.9%
Bisexual, Bisexual/Queer Only	94	26.2%
Pansexual, Pansexual/Queer Only	44	12.3%
Other (Bi/Pan/Queer, etc.)	125	34.8%
Missing	14	
Gender Identity^d^		
Man Only	85	22.7%
Woman Only	155	41.6%
Other (genderqueer, nonbinary, trans, etc.)	133	35.7%
Missing	0	
Relationship Identity		
Monogamous	88	25.0%
Nonmonogamous	264	75.0%
Polyamorous	203	57.7%
Missing	21	

Data from Stage 2 of *The Pleasure Study* (Harvey et al., 2023), *N* = 373

^a^Respondents were able to choose multiple race/ethnicities

^b^On the survey the race/ethnicity options were labeled American Indian/Alaskan Native and Native Hawaiian/Pacific Islander

^c^Respondents were able to choose multiple orientations

^d^Respondents were able to choose multiple gender identities

For the current study, I included all survey participants from the sample described above who indicated on the survey previous attraction to a couple. The sample, a total of 145 survey participants (38.9% of *The Pleasure Study* survey participants), included a large percentage of queer sexualities (90.3%) and genders (34.5%), as well as nonmonogamous relationship identities (87.5%). Participants were predominately between 21 and 40 years old (74.4%), White (66.4%), not religious (78.0%), attained a bachelor’s degree or higher (71.1%), middle class (47.4%), living in the USA (65.5%), and living in urban areas (83.7%). See Table 2 for the demographic distributions of the study sample.

**Table 2:** Survey respondents who experience symbiosexual attraction

Variable	Frequency	Valid percentage
Age (in years)		
21–30	43	33.3%
31–40	53	41.1%
41–50	20	15.5%
51–60	11	8.5%
61–70	2	1.6%
Missing	16	
Race/Ethnicity^a^		
Asian/South Asian	6	4.4%
Arab	0	0.0%
Black/African American	15	11.0%
Hispanic/Latinx/Spanish	12	8.8%
Indigenous Peoples^b^	8	4.8%
Jewish	10	7.3%
Middle Eastern/North African	0	0.0%
White (Only)	91	66.4%
Missing	8	
Religion		
Important	29	22.0%
Christian/Catholic	9	
Jewish	5	
Pagan/Witchcraft	10	
Not Important	103	78.0%
Missing	13	
Education		
Less than High School	0	0.0%
High School/GED	2	1.5%
Some College/Associates	37	27.4%
Bachelors	45	33.3%
Masters	41	30.4%
Doctoral/Professional	10	7.4%
Missing	10	
Social Class		
Working	48	36.1%
Middle	63	47.4%
Upper/Middle, Upper	22	16.5%
Missing	12	
Country/Region		
USA (29 States)	78	65.5%
Mid-West	19	
North-East	16	
South-East	20	
South-West	5	
West	18	
Outside USA (9 Countries)	41	34.5%
Australia	1	
Canada	30	
Germany	2	
India	1	
Ireland	1	
Luxembourg	1	
Peru	1	
Portugal	1	
UK	3	
Missing	26	
Community Type		
Rural	22	16.3%
Urban	113	83.7%
Missing	10	
Sexual Orientation^c^		
Asexual Only	1	0.7%
Heterosexual Only	14	9.7%
Gay, Gay/Queer Only	8	5.5%
Lesbian, Lesbian/Queer Only	4	2.8%
Bisexual, Bisexual/Queer Only	34	23.4%
Pansexual, Pansexual/Queer Only	26	20.0%
Other (Bi/Pan/Queer, etc.)	58	40.0%
Gender Identity^d^		
Man Only	33	22.8%
Woman Only	62	42.8%
Other (genderqueer, nonbinary, trans)	50	34.5%
Relationship Identity		
Monogamous	18	12.5%
Nonmonogamous	126	87.5%
Polyamorous	97	67.4%
Missing	1	

Data from Stage 2 of *The Pleasure Study* (Harvey et al., 2023), *N* = 145

^a^Respondents were able to choose multiple race/ethnicities

^b^On the survey the race/ethnicity options were labeled American Indian/Alaskan Native and Native Hawaiian/Pacific Islander

^c^Respondents were able to choose multiple orientations

^d^Respondents were able to choose multiple gender identities

I also included data from all interviewees who indicated on *The Pleasure Study* survey previous experience of attraction to a couple. The sample, a total of 34 interviewees (81.0% of *The Pleasure Study* interviewees), included diverse ages, race/ethnicities, religions, social classes, nationalities, regions, community types, sexualities, genders, relationship identities. See Table 3 for individual interviewee demographic information.

**Table 3:** Survey data of individual interviewees who experience symbiosexual attraction

Pseudonym	Demographic data					
	Gender	Sexuality	Relationship identity	Race	Age	Location
Amari	Nonbinary	Queer/skoliosexual	Polyamorous	White	39	Georgia (urban)
Angel	Nonbinary/woman/dyke	Bisexual/lesbian/queer	Polyamorous	White/Jewish	26	Illinois (urban)
Asa	Woman	Bisexual/pansexual	Polyamorous	Hispanic/White	32	Texas (urban)
Avery	Trans man	Queer	Monogamous	White	28	Michigan (urban)
Bellamy	Nonbinary/trans/femme	Bisexual/gay/pansexual/queer	Polyamorous	Asian/White	27	Mississippi (rural)
Blake	Man	Gay	“Currently monogamous”	White	28	Michigan (urban)
Cameron	Man	Asexual/gay/queer	“Currently Nonmonogamous”	White	35	Ohio (urban)
Casey	Trans woman	Bisexual/pansexual/queer	“Preferably monogamous”	White	missing	Iowa (urban)
Charlie	Woman	Pansexual	Polyamorous	White	42	California (rural)
Devin	Man	Heterosexual/heteroflexible	Nonmonogamous	White	32	California (urban)
Drew	Man	Heterosexual	Polyamorous	White	45	Illinois (urban)
Eden	Man/“want to be genderfluid”	Bisexual/pansexual/queer	Polyamorous	White	30	Illinois (urban)
Ellis	Nonbinary/woman	Bisexual/pansexual/queer	Polyamorous	White	33	Massachusetts (urban)
Harlow	Nonbinary/trans	Queer	Polyamorous	Jewish/White	34	New York (urban)
Hayden	Woman	Queer	Ethically nonmonogamous	White	52	Canada (urban)
Kamari	Man/bigender	Queer/polysexual	Polyamorous	Black (Caribbean)	50	Canada (urban)
Kendall	Nonbinary/woman	Bisexual	Monogamous	White	27	Indiana (rural)
Lennon	Nonbinary/genderqueer	Queer	Ethically nonmonogamous	White	30	New York (urban)
Logan	Woman	Bisexual/queer	Monogamous	Hispanic	23	Florida (rural)
Noa	Nonbinary	Queer	Polyamorous	White/Jewish	25	Oregon (urban)
Onyx	Nonbinary	Bisexual	Polyamorous	White	39	UK (rural)
Parker	Nonbinary	Pansexual/queer	Ethically nonmonogamous	White	36	California (urban)
Phoenix	Woman	Bisexual	“Situationally”monogamous/Polyamorous	White	23	Germany (urban)
Peyton	Woman	Bisexual	Ethically nonmonogamous	Asian/White	30	California (urban)
Quinn	Nonbinary	Lesbian	Polyamorous	White	23	Kentucky (urban)
Reece	Woman	Bisexual	Polyamorous	White	48	Canada (urban)
Riley	Woman	Pansexual	Polyamorous	White	55	Canada (urban)
River	Nonbinary/trans man	Pansexual/queer	Polyamorous	White	34	Virginia (urban)
Rowan	Man	Pansexual	Consensually nonmonogamous	White	59	Minnesota (urban)
Sage	Nonbinary	Pansexual	Polyamorous	White	39	Missouri (urban)
Sawyer	Woman	Bisexual/queer	Ethically nonmonogamous	White	35	Luxembourg (urban)
Skyler	Woman	Heterosexual	Ethically nonmonogamous	Asian	40	Canada (urban)
Taylor	Trans woman	Sapphic bisexual	Polyamorous	White	missing	Oklahoma (urban)
Teagan	Man/nonbinary/transman/woman/Nonconforming	Pansexual/queer	Polyamorous	Indigenous Peoples^a^/White/“Culturally multicultural, mostly black”	49	Illinois (rural)

Data from Stage 2 of *The Pleasure Study* (Harvey et al., 2023), *N* = 34

^a^On the survey the race/ethnicity options were labeled American Indian/Alaskan Native and Native Hawaiian/Pacific Islander

### Measures and Procedure

*The Pleasure Study* survey instrument consisted of 65 questions and took participants approximately 20 min to complete online. The survey included a mix of multiple choice, Likert scale, and open-ended questions about gender, sexual orientation, relationship practices, culture, education, performance of sexual pleasure and specifically included questions about experiences with couples. The study also included interviews with those who indicated (in the survey) willingness to participate in an interview. Researchers conducted interviews over Zoom, lasting between 1 and 2 h. Participants answered semi-structured questions about their gender, orientation, relationship practices, culture, education, and performance of sexual pleasure, as well as questions about romantic and sexual experiences with couples. Audio files recorded via Zoom were transcribed using OtterAI Pro transcription software. Transcripts were cleaned and anonymized by myself (as the research coordinator for *The Pleasure Study*) and *The Pleasure Study* research assistants.

I conducted an integrated mixed-methods analysis (Creswell et al., 2011; Fetters et al., 2013) of the secondary data from *The Pleasure Study.* Creswell et al. (2011) and Woolley (2009) recommend using integrated mixed-methods when examining an unstudied phenomenon like symbiosexual attraction because it offers a way to use multiple sources of information to affirm or challenge novel findings and because with unstudied phenomena it is unknown prior to analysis what kinds of data may offer the most useful information. Further, this method offers more breadth and depth of information for topics that lack data—reductive information that can be used to succinctly describe a phenomenon and holistic information that provides a more complete picture of the nature of the phenomenon (Woolley, 2009). Combining qualitative and quantitative data in analysis can enrich understanding of the nature of a phenomenon like symbiosexuality, where it surfaces, and how it operates (Creswell et al., 2011).

For this study, I analyzed data using a convergent parallel design, a basic integrated mixed-methods design that considers qualitative and quantitative data equally in analysis (Creswell et al., 2011). I selected this design because it is appropriate for secondary data as qualitative and quantitative data do not need to be combined prior to analysis and because by examining together and comparing qualitative and quantitative I could more fully examine the nature of the complex phenomenon of symbiosexual attraction (Fetters et al., 2013). By combining and comparing the data I was also able to investigate the ways in which quantitative and qualitative data complemented or contradicted one another (Fetters et al., 2013).

I used a convergent parallel design for integrated mixed-methods analysis. I analyzed survey and interview data sets separately and then combined data sets to produce findings that would more thoroughly answer my research questions and to compare quantitative and qualitative information for consistencies that would further support my conclusions or highlight discrepancies that would need further investigation.

For the purpose of this study, I operationalized symbiosexual attraction as the experience of attraction to a couple. All participants included in the study answered yes on the survey to the question: Have you ever felt sexually/romantically attracted to a couple (two people and their relationship together, not each of them individually)? Survey questions about the frequency of this attraction, demographic questions (gender, sexual orientation, relationship identity, race, religion etc.) (see Appendix A), and interview questions about the experience of attraction to couples were included in analysis (see Appendix B).

Key survey questions included:

1. “Have you ever felt sexually/romantically attracted to a couple (two people and their relationship together, not each of them individually)”?
2. “Regarding your attraction to couples (two people in a relationship together) I have experienced this attraction…”

Participants answered Question 2 using a Likert-type scale from 1 to 5 (never to often).

The key interview questions included:

“You indicated on the survey that you experience attraction to couples, when you have experienced this attraction, what about the couple(s) was attractive to you?”

### Data Analysis

I analyzed descriptive statistics and frequency tables derived from quantitative data using SPSS Version 28. I analyzed qualitative data from the interviews for thematic content relevant to the nature and experience of symbiosexual attraction. Using thematic analysis (Bryman, 2016), I organized qualitative data by interviewee descriptions of their personal characteristics, descriptions of what they were attracted to with couples, and descriptions about how they experienced this attraction. For each of these subtopics, I grouped descriptions until themes emerged. Eight themes emerged from interviewee descriptions of personal characteristics, five themes emerged from interviewee descriptions of what they were attracted to with couple, and 2 themes emerged from interviewee descriptions about how they experienced symbiosexual attraction. Once I analyzed quantitative and qualitative data sets separately, I examined the data sets together and compared them to answer my research questions.

## Results

### Do People Experience Symbiosexual Attraction? If So, What Are Their Demographic and Personal Characteristics?

I found strong evidence of symbiosexual attraction by combining and comparing survey and interview data. Of the 373 Pleasure Study survey participants, 145 (38.9%) reported experiencing attraction to people in a relationship, specifically couples. All who reported experiencing this attraction on the survey, confirmed during the interview that they felt this attraction (*n* = 34).

I found breadth and depth of information on the profiles of people who experience symbiosexual attraction by combining data sets. Profiles included demographic descriptors as well as personal characteristics of people who experience this attraction.

### Demographics

The 145 survey participants who reported experiencing symbiosexual attraction represented a diverse group of ages, race/ethnicities, religious beliefs, education levels, social classes, nationalities, US regions, community types, sexualities, gender identities, and relationship identities (see Table 2). They were predominately between 21 and 40 years old (74.4%), White (66.4%), not religious (78.0%), attained a bachelor’s degree or higher (71.1%), middle class (47.4%), living in the USA (65.5%), and living in urban areas (83.7%). A large percentage of participants represented queer sexualities (90.3%) and genders (34.5%), as well as nonmonogamous relationship identities (87.5%) (see Table 2).

### Personal Characteristics

The 34 participants who completed the interview described unique personal characteristics that they felt explained why they feel symbiosexual attraction. These characteristics included extroversion, wanting lots of intimacy, care, and/or validation, not experiencing jealousy, being compersive,^1^ nonmonogamous preferences, and sexual openness and queerness. An example of an interviewee attributing a characteristic of themselves to their attraction to couples was Angel who explained that they are attracted to couples because they “like a lot of care and affection and intimacy in my sex. So [being with couples] brings a lot of that upfront.” Another example was Eden who explained that he was attracted to couples because “I have this desire to be desired and I seek a lot of validation, a lot of validation, and when there are multiple people like that, I feel like oh, yes, yes, I’m doing things right.” A third example was Charlie who explained that she is attracted to couples because she is “super compersive” and specifically drawn to couples who have built strong, secure relationships and can share in that compersion. Compersion has been studied in recent years as a personal characteristic of people who identify as nonmonogamous (Flicker et al., 2022). This interviewee’s description suggests it may also be found in those who experience symbiosexual attraction.

The large number of people from *The Pleasure Study* reporting symbiosexual attraction and the diversity of demographic profiles and personal characteristics of those who experience this attraction indicates that this attraction exists and can be found in a variety of populations. Further interviewee descriptions of personal characteristics they attribute to symbiosexual attraction suggests that some who experience this attraction have full awareness of the attraction that is paired with strong self-awareness about their unique and in some cases less normative personal characteristics and/or preferences (such as not experiencing jealousy or preferring nonmonogamous, open sex and relationship dynamics). In these descriptions, interviewees went beyond describing motivations for being attraction to couples, they highlighted core truths about themselves which they felt explained why they experience this attraction.

### How Do People Describe Symbiosexual Attraction? To What Relationship Dynamics Are They Specifically Drawn?

By combining quantitative and qualitative data, I found that many people of diverse identities and backgrounds (as reported on the survey) describe a similar experience of symbiosexual attraction in their interview: a distinct and specific attraction to relationships between people. Their interview responses provide information on how people describe symbiosexual attraction, how this attraction is distinct from an attraction to individual people, and the specific characteristics of relationship dynamics to which people who experience symbiosexual attraction are drawn.

### Symbiosexual Attraction

When asked what they found attractive about couples, interviewees described a draw to what happens between and emanates from people in an established relationship. Interviewees specifically described their draw to the energy, cohesion, charisma, multidimensionality, and power created by couples.

Many interviewees highlighted their attraction to the energy couples emit through their interactions and engagements. Displays of cohesion and unity created a perception of synergy people with symbiosexual attractions found appealing and intriguing. Hayden explained what she found attractive about couples was “their cohesiveness, you feed off their energy, their attraction to each other…there’s an interplay between the couple.”

The perception of positive synergy also functioned as an enticing entry point for interviewees who wished to be “adopted” or immersed into a dynamic that seemed safe and inviting. Sage described remembering developing feelings toward a specific couple, “I also just want to be smack in the middle of that relationship. I would also like to be included in this relationship… I really think my ideal dynamic might be myself and a couple.” Tentatively sharing this perspective, Ellis explained:

It’s just that much more intriguing and attractive to think of huh, what would it be like if I were in that part of their relationship or if I ended up being brought into their relationship? What would that be like? You know this would be like really great… seeing the way they interact, in the way that they sort of have a have a rapport with each other.

As if offering an explanation for Ellis’s trepidation of acting on this desire, another interviewee, Cameron questioned whether his desire to immerse himself in an established relationships was healthy:

I think to some degree, as a single man, there’s a certain attractiveness to, to a relationship, to people in a relationship. Whether or not that’s psychologically healthy, I think we can debate a little bit... I definitely am attracted to that partnering, to that coupling, to even that love between two people. And perhaps on some level, wanting to participate in that, in some way, shape, or form.

Cameron seemed to grapple with whether his desire is a yearning for a relationship or a yearning for people in relationship, suggesting that he considers the former “healthy” or “normal” but is uncertain about the latter.

Participants also described an attraction to the specific chemistry couples created and displayed through their interactions: a heightened charisma to that offered by individuals. Peyton described an attraction to the “in sync-ness” between a couple, she elaborated on this by explaining her attraction to one particular couple: “They are very fun and flirty and sexy together and they’re just like very charismatic as a unit.”

Participants also found the multidimensionality offered by couples appealing. The complex web of shared experiences, emotions, and histories between couples added depth and richness to their appeal, compelling observers into their world. Further, beyond their individual characteristics and experiences, couples exhibited complementary attributes including appearances, personality traits, and different energies or identities that for participants synthesized in an irresistible way. Kamari described how “delicious” couples are when their different yet complementary “energies are flowing together.”

Participants also found the power created by a couple enticing. They spoke about attraction to the collective strength that arises from people in relationships: the power built through mutual support, shared goals and desires and complementary individual strengths. Some participants felt desire to draw on this power source, while others wanted to succumb to it. In either case, interviewee descriptions acknowledged that the power created through people in a relationship is more than two individual energies coming together. This was exemplified by Parker who described their attraction to one specific couple:

The combination of them is just--it’s a transcendent thing. It’s beautiful and they have one of the most beautiful relationships that I know ... it is definitely very much not just about the sum of the parts, but something that is greater than that. There’s something synergistic.

Parker likened their attraction to attractions they had heard other’s express about them and their current partner. They explained that others were drawn to “the powerful dynamic, essentially, of our complementary energies.” Here they acknowledge that the power of two people in relationship is not a simple formula of addition but of multiplication, something stronger and more appealing both as the observer of couples and as a potential creator of this power in partnership.

*Not Symbiosexual Attraction* Of the 34 interviewees, two described experiences of attraction that would not be labeled as symbiosexual. While both interviewees reported that they experience attraction to couples as a unit on the survey, in the interview they described this experience of attraction as more of a serendipitous event where they just so happened to like both members of the couple. Onyx explained, “I think I liked them both as individuals…attraction is the wrong word, I think I was just in a really lucky situation.” Kendall explained:

There have been maybe one or two couples throughout my life where I just really, really liked both of the people in the couple and at least one of them I was physically or sexually attracted to…it’s more about just liking both of the people independently and respecting that relationship.

These descriptions reflect more of an attraction to individual people that happened to be in a relationship than an attraction to the dynamic of the couple.

In sum, interviewees who were attracted to couples were notably attracted to the intricate layers and nuances that emerge when two individuals come together, forming a relationship that is more the sum of its parts. Their descriptions echo the findings in my examination of discourse surrounding attraction to relationships (Johnston, 2023). There is a common thread of attraction to a “third force” that surpasses the mere combination of two individuals. This concept captures the essence of the attraction experienced by individuals toward couples, emphasizing the allure of the synergistic energy and power that arises when two people come together in romantic partnership.

### Attractive Characteristics of Relationships

For the 32 interviewees who described attraction to the dynamic between couples, certain characteristics of relationship dynamics were mentioned multiple times. Five themes emerged in these descriptions including the couples’ intimacy, relationship quality, physical appearance, level of playfulness, and inherent gender and/or sexual queerness.

*Intimacy* Interviewees specifically talked about being attracted to the built intimacy offered through people in an established relationship. Angel explained that in dynamics with couples, “You’re already working within this field of intimacy that’s there already. And so, you don’t have to establish it.” Echoing this, Sawyer explained that she specifically enjoyed “the sensation of being invited inside the intimacy” that couples share. Adding an element, Lennon explained, “It’s really interesting to dip in and out of a pre-existing relationship…to see intimacy both from the inside and outside.” These descriptions suggest that some people may specifically be drawn to a pre-established or multidimensional experience of intimacy uniquely offered through sexual and romantic engagement with people in relationships.

*Relationship Quality* In describing the experiences of attraction to the dynamic between couples many interviewees highlighted feeling attracted to relationships that were strong, loving, healthy, and had great communication. As Ellis explained, “There's something really nice to me about saying okay, well, these two people already know how to be in a relationship in a really healthy and great way and that’s attractive.” Asa shared this perspective:

If your perception is that they have a healthy, good relationship, then yeah what’s not attractive about someone who’s good to their partner and communicates well and is caring? Those are good things.

Asa’s attraction to partner care seems to overlap with the concept of compersion described in the section on personal characteristics.

Interviewees also specifically mentioned communication skills as a feature of quality relationships. Avery explained:

I think for me, it’s really about outward communication. I grew up in a family that doesn’t communicate very well, we still don’t communicate very well, so I feel like when I see couples that have that, they’re really strong and on the same page, and really understand each other’s communication styles, I feel like that’s the biggest thing. I’m like, wow they’re good.

Similarly, Phoenix explained of a couple she was attracted to:

They are quite open in their communication with each other, which I just find really desirable in a relationship. And also, the way they think a lot about the other person when they make decisions, for example, or them always being very considerate, the way they interact with each other.

One interviewee, Rowan described a healthy relationship as a criterion for the couples he was attracted to, “There has to be a healthiness between the two of them … mutual respect among everyone.” Good communication was an indicator of health in the relationship of the couple that he was currently attracted to. He explained, “The communication is amazing. That is something that is really, really great.” These interviewees seemed to be drawn to the assurance that there is a healthy dynamic offered through established couples which may in some case be motivated by the opportunity to safely explore sexual and romantic dynamics or safely repair past experiences that they felt were unhealthy in some way.

*Physical Appearance* In describing the experiences of attraction to the dynamic between couples some interviewees highlighted feeling attracted to the appearances of the couple—their vibe and how they look as a unit. Lennon highlighted this when they explained how they experience attraction to couples:

I experienced this attraction first on a purely physical level. I think that there are times where people just aesthetically look really good together so that’s hot. And then also especially any sort of alternative couples like, great.

Similarly, Skyler explained that what she is attracted to with couples is “How they look together … I like how they look together, and I feel like they would be a good fit, physically for myself.” Peyton described her experience of being physically drawn to a specific couple “I was like, wow, both her and her husband are really hot. It was wild, like both? Rarely do you see a completely matched couple in terms of attractiveness.” The implication of these descriptions is that there was interplay between the look of each member of the couple that added to their appearance as a unit whether that look created an alternative vibe, something complementary or matching, or just heightened the physical appeal.

*Playfulness* In describing their experiences of attraction some interviewees highlighted being drawn to the fun possible in a playful dynamic between the couple. Citing fun as a criterion for symbiosexual attraction, Casey explained there has to be “a certain level of playfulness” to the dynamic. Others elaborated on an element of playfulness that drew them in. Harlow explained that it was the play of their sexual connection: “Three people is really, really, really fun. And when two people have a good sexual chemistry, and then they invite you in that’s really fun.” Angel explained that it was the playing out of love between people that was attractive, “I think it’s really nice to obviously be with two people that are hot and fun. But it’s also fun to watch two people who care about each other express that.” Another interviewee characterized a specific couple as fun and playful. Reece explained of a specific couple they were attracted to, “I thought they were really fun people and I just wanted to see what it’d be like being together.” These interviewees seemed to be talking about a desire to join the party certain couples create in their relationship dynamic.

*Queerness* In describing their experiences of attraction some interviewees described being drawn to the inherent sexual and/or gender queerness of dynamics with couples. Explaining how their attraction to couples was affirming, Eden, who identifies as bisexual/pansexual/queer and who was curious about gender fluidity, explained that his desire for couples, if mutual,

validates a lot of my queer identity of, oh great, people of different genders are attracted to me. And that's not the only way it goes because then if they’re of the same gender then I am like oh well, they’re also queer, too. I have a queer acceptance…I think it’s just this freeing that it can be something different than what we traditionally have been taught. It’s that absence of rules, of expectations. I am very excited by different possibilities.

Also speaking to the expanded possibilities offered through couples, Rowan explained why couples are desirable for him:

It’s because the gender sort of falls away and that’s really what I find attractive about it. And it’s not that it’s not masculine or feminine energy that’s there. It’s just that doesn’t matter. Even the emotional part is really beautiful, because it seems to transcend gender. Yeah, and that’s what I that’s sort of what I like about being a submissive and why I’m attracted to kind tops that are that are often lesbian looking or bisexual…sort of the gender fuckery of all that.

The idea that two people in relationship offer a different experience of gender was shared by Kamari, who described his attraction specifically to “strong masculine and feminine energy bound together” in the unit of the couple.

Some interviewees talked about specific gender combinations that they were drawn to. Angel explained that they have found themselves specifically attracted to a couple where both partners are nonbinary and explained how sex with two people is great because it is “inherently a little … I was gonna say goofy or awkward or not normative, but you’re already coming in being like well we’ll see what this is gonna be.” Also addressing the desirability of multiple genders, Charlie explained that couples are particularly attractive “when it’s two genders, like when you have a man and a woman. I’ve also had interactions with a cis man and a trans woman, and gosh, that was, that was so much fun.” She goes on to explain that it was fun because “even though they’re both really queer, they both have super different energy.” The dance of the energies between two people, whether of different gender identity or not, ignited a desire that interviewees recognized as queer: a desire that for some offered more flavors and possibilities for diverse sexual and gender expression.

Interviewee descriptions of couples’ characteristics that they are specifically attracted to adds depth to our understanding of symbiosexual attraction and affirms that this attraction is a draw to the complexity and amplification of factors like intimacy, relationship quality, physical appearance, level of playfulness, and gender and/or sexual queerness. People in relationships display these factors with a strength and multidimensionality that is not possible in one-to-one dynamics.

### How Significant Is Symbiosexual Attraction in People’s Lives?

By combining and comparing survey and interview data, I found information on how symbiosexual attraction is experienced by individual people including the frequency and strength of the attraction, as well as how people become aware of this attraction. Survey responses provided information on how frequently individual people experience symbiosexual attraction. Interview responses highlighted the strength of the experience of symbiosexual attraction for some people. Comparing data sets, 13 of the 34 interviewees described experiences of symbiosexual attraction that were strong, significant, and/or pervasive. The experiences were documented either by the reported frequency of their attraction on the survey, their enthusiastic interview descriptions, or both.

### Frequency of Symbiosexual Attraction

**Fig. 1:**
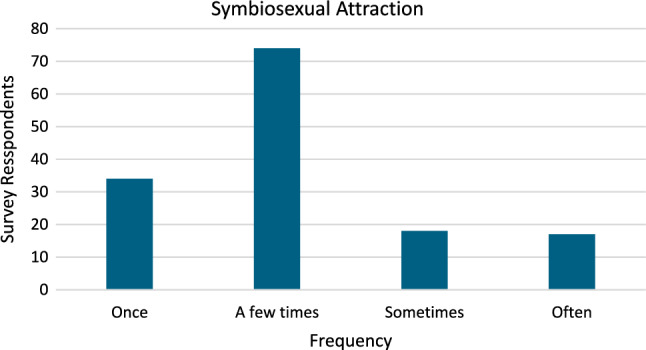
Frequency of symbiosexual attraction. *Note* Data from Stage 2 of *The Pleasure Study* (Harvey et al., 2023). *N* = 143

Thirty-five survey participants (24.5%) reported experiencing attraction to couples sometimes or often indicating that for some people symbiosexual attraction is a frequent and/or pervasive experience. Seventy-four survey participants (51.7%) reported experiencing attraction to couples a few times which may or may not be evidence of symbiosexual attraction as a significant lived experience. Thirty-four survey participants (23.8%) reported experiencing attraction to couples once which may be evidence of a one-off experience less relevant to the lived experience of their sexuality (see Fig. 1).

### Enthusiasm About Symbiosexual Attraction

In addition to the reported frequency of symbiosexual attraction, I found evidence of the potential strength of this attraction through interviewee enthusiasm. On some occasions, the interviewer asked a question confirming that the participant experienced attraction to couples, as they indicated on the survey. All who were asked, as well as all those who were not directly asked, confirmed they experienced this attraction. For some participants, however, their responses were notably enthusiastic when asked to confirm that they experience attraction to couples. For example, when asked if they experienced this attraction Bellamy responded “Yeah! Yeah.” They went on to explain that nonmonogamous dynamics such as those with couples are “very much a part of who I am and how I work.” Similarly, Lennon responded with an empathic “Oh, yeah” and went on to explain they are “very attracted to couples” and delighted in the power of being responsible for other people’s experimentation which is inherent when you enter a couple dynamic. In another example, Amari gave a long empathic “MMMHMMM” response. They attributed that response to their “extreme” extroversion which was complimented by the social abundance offered through couples. Finally, Rowan, who described sex with a couple as his “favorite sexual experience” and “preferred sexual encounter,” responded “Oh yeah!” when asked about the attraction. He confessed it gave him “warm feelings just thinking about it.” These emphatic responses were all punctuated by interviewee explanations that their attraction to couples was a central, primary, and/or very strong lived experience for them.

### An Unfamiliar and Unexpected Attraction

Regardless of the frequency or strength of their symbiosexual attraction, interviewees explained that feeling this attraction was initially novel, surprising, and unexpected. Despite broad and eclectic identifications (including multiple races, genders, and sexualities), Teagan described their surprise and intrigue at feeling symbiosexual desire for the first time,

I met these people, and it was very interesting because instead of viewing them as two individual people, I saw them as one entity … I had never experienced anything like that before… I never actually experienced different people as one as a unit ... it was just totally different.

In another example of coming upon this attraction by surprise, Rowan explained that his interest in couples began when he “bought a couple of swingers magazines” and found himself specifically turned on by couples. He said, “I didn’t think it would be couples, I was surprised that it was couples.” Similarly, Taylor described the novelty and surprise of the experience: “It was an interesting attraction to have built. It was something new. I’d never experienced it before that point … a little bit harder to quantify, though, than attraction to the individuals themselves.”

Other interviewees found this experience difficult to describe. Some interviewees talked specifically about not having words or language for the attraction they felt. Onyx explained:

It was 2002, so we didn’t have the words or the language or really know what we were doing. We’re just like, oh, we all want to go to the same place … it crossed my mind, like, oh I’m supposed to fancy one person and I don’t. I think they’re both really wonderful human beings. And I remember thinking, how does that work? ... It takes knowing words to be able to think sometimes and we didn’t have those words and those language so a lot of it was just trial and error and running through.

Also struggling for words, Sage recalled the first time they felt attracted to a couple: “I was like, what is this feeling?.”

One interviewee, Logan talked about being surprised to learn that her experience of attraction was not typical. “I used to think that that’s how it was for everybody. And I was talking to my friend and then I realized that’s not how it is for everybody. Not everybody experiences this attraction to groups like that.” Another interviewee, Peyton talked about not being aware that wanting couples was even possible:

I definitely had fantasies or thought about like, what if we could all be in a relationship together? Like, wouldn’t that be magical if we could all just be together? It always felt like it couldn’t happen or that it was just too wild to even think about … I didn’t even know that this was an option.

Interviewees were able to recognize their desire but, because of its cultural invisibility and lack of language, they found it difficult or beyond the realm of possibility to entertain.

Despite the unexpected nature of this experience interviewees were able to recall their initial and growing awareness of this attraction and the accompanied (and understandable) uncertainly of such an experience. Interviewees struggled both with how to contextualize the experience as well as what, if anything, should be done about it. Without language like the term *symbiosexual* and without validation or recognition of multidirectional, multidimensional attractions interviewee descriptions revealed a gap between their awareness of this desire and the labels and behaviors that may affirm it.

## Discussion

There were several key findings from this study. First, I found that the phenomenon of symbiosexual attraction exists and is experienced by a diverse group of people. Second, I found that symbiosexual attraction is as an attraction to a variety of relationship dynamics between people in preexisting relationships. Third, I found that, while unexpected or unfamiliar, symbiosexual attraction can be a potentially significant experience. These findings reveal important information about the existence and nature of symbiosexual attraction which challenge ongoing symbiosexual invisibility, invalidation, stigma, and discrimination. These findings also challenge current conceptions of desire and attraction in sexuality studies.

### Symbiosexual Attraction Exists and Is Experienced by a Diverse Group of People

A large number of affirmative survey and interview responses about attraction to couples support H1 that some people experience attraction specifically to the relationships between people. While queer, nonmonogamous, and people who experience attraction to couples were specifically recruited, it was surprising that so many people (38.9% of *The Pleasure Study* sample) reported experiencing symbiosexual attraction. Survey data also supported H1 that people of diverse ages, race/ethnicities, religious beliefs, education levels, social classes, nationalities, US regions, community types, sexualities, gender identities, and relationship identities experience symbiosexual attraction. These findings suggests that symbiosexuality is a traceable lived experience for a diverse population.

It should be noted that a large percentage of participants reported queer sexualities, genders, and relationship identities. In addition, participants were predominantly between 21 and 40 years old, White, not religious, attained a bachelor’s degree or higher, middle class, living in the USA, and living in urban areas. These findings are reflective of the skew of the larger Pleasure Study sample and, due to recruitment methods, are not generalizable. More research is needed on how those who experience symbiosexual attraction compare to the general population.

A significant percentage of survey participants reported plurisexual and nonmonogamous relationship identities, including bisexual and pansexual identities, which also supports H1. Interestingly, while many women reported symbiosexual attraction (supporting H1), a surprisingly large number of people who experience attraction to couples in this study identified outside the gender binary. While the high percentage of non-normative sexuality, relationship, and gender identities reporting symbiosexual attraction makes sense in relationship to a non-normative attraction, it is unknown if those who experience this attraction are more likely to identify in these ways. The primary recruitment efforts in *The Pleasure Study* toward queer and nonmonogamous populations likely skewed these results. It should be noted that those who reported experiencing symbiosexual attraction on the survey differed in two meaningful ways from the portion of *The Pleasure Study* population who reported not experiencing symbiosexual attraction or being unsure if they have had this experience. This latter population included a higher rate of heterosexuality (11.2%), a lower rate of pansexuality (− 10.8%), and a lower rate of nonmonogamy (− 21.2%) (see Table 4). These differences are unsurprising, but more research is needed on symbiosexuality to understand if those who experience this attraction are more likely to choose queer gender and sexuality labels.

**Table 4:** Survey respondents who do not experience symbiosexual attraction (or not sure)

Variable	Frequency	Valid percentage
Age (in years)		
21–30	80	44.4%
31–40	61	33.9%
41–50	32	17.8%
51–60	5	2.8%
61–70	1	0.6%
71–80	1	0.6%
Missing	26	
Race/Ethnicity^a^		
Asian/South Asian	11	5.6%
Arab	0	0.0%
Black/African American	24	12.2%
Hispanic/Latinx/Spanish	17	8.6%
Indigenous Peoples^b^	4	2.0%
Jewish	13	6.6%
Middle Eastern/North African	2	1.0%
White (Only)	131	66.5%
Missing	9	
Religion		
Important	54	27.8%
Christian/Catholic	18	
Jewish	10	
Pagan/Witchcraft	6	
Not Important	140	72.2%
Missing	12	
Education		
Less than High School	1	0.5%
High School/GED	12	6.1%
Some College/Associates	47	23.9%
Bachelors	59	30.0%
Masters	61	31.0%
Doctoral/Professional	17	8.6%
Missing	9	
Social Class		
Working	70	35.5%
Middle	102	51.8%
Upper/Middle, Upper	25	12.7%
Missing	9	
Country/Region		
USA (38 States)	140	74.1%
Outside USA (14 Countries)	49	25.9%
Missing	17	
Community Type		
Rural	32	16.3%
Urban	164	83.7%
Missing	10	
Sexual Orientation^c^		
Asexual Only	5	2.4%
Heterosexual Only	43	20.9%
Gay, Gay/Queer Only	14	6.8%
Lesbian, Lesbian/Queer Only	14	6.8%
Bisexual, Bisexual/Queer Only	60	29.1%
Pansexual, Pansexual/Queer Only	19	9.2%
Other (Bi/Pan/Queer, etc.)	51	24.8%
Gender Identity		
Man Only	43	20.9%
Woman Only	82	39.8%
Other (genderqueer, nonbinary, trans)	81	39.1%
Relationship Identity		
Monogamous	69	33.7%
Nonmonogamous	136	66.3%
Polyamorous	105	
Missing	1	

Data from Stage 2 of *The Pleasure Study* (Harvey et al., 2023), *N* = 206. Respondents were able to choose multiple race/ethnicities

^a^Respondents were able to choose multiple race\ethnicities

^b^On the survey the race/ethnicity options were labeled American Indian/Alaskan Native and Native Hawaiian/Pacific Islander

^c^Respondents were able to choose multiple orientations

Interviewees attributed personal characteristics of themselves to their symbiosexual attraction including extroversion, wanting lots of intimacy, care, or validation, not experiencing jealousy, being compersive, nonmonogamous preferences, and sexual openness and queerness. Their descriptions evoke a “born this way” narrative not unlike those with other queer attractions. More research is needed on whether people who feel such attractions experience them as innate, core attractions that may be associated with their identity and/or orientation. Whether experienced innately or not, their descriptions suggest that certain kinds of people may be more likely to desire sex and relationships with couples.

The mention of the characteristic of compersiveness to explain symbiosexual attraction is specifically intriguing. Flicker et al. (2021) identified “erotic feelings toward an existing partner-metamour relationship” as a factor that facilitates the lived experience of compersion (p. 1577). This factor of compersion, which Flicker et al. label as Sexual Arousal on the scale they develop to measure compersiveness, directly describes symbiosexual attraction (sexual attraction to a preexisting couple). People within nonmonogamous communities who describe themselves as compersive because they experience this arousal/excitement toward witnessing and engaging with people in relationships could also, alternatively, or additionally be describing symbiosexuality. Research on the connection between compersion and symbiosexual attraction is needed.

### Symbiosexual Attraction Is an Attraction to a Variety of Relationship Dynamics Between People

Interviewee descriptions of their attractions provides rich information about the nature of symbiosexual attraction. These descriptions from people of very diverse backgrounds and identities support H2 that symbiosexual attraction is an attraction to a variety of relationship dynamics between people; the cohesiveness, charisma, energy, multidimensionality, and power made possible through relationships. While not describing a tangible object, interviewees shared the perception of this “third force” created by people in relationship, as described by Giuliani (2021) in her experiences with couples. The existence of this force was previous identified by research scholar Wade (2004) in her examination of transcendent sexual experiences between people. Wade found that two people, in their “mutuality,” create a “force” or “third presence” (p. 273). Attraction to this force between people is distinct from multiple individual attractions. As such, more mainstream orientation labels like bisexual or pansexual are insufficient to describe this phenomenon. The term and concept of symbiosexuality, as a multidirectional, multidimensional experience of attraction, needs attention and recognition.

Interviewees not only expressed awareness of this unique attraction but were able to articulate specific features of relationships that they were drawn to including intimacy, relationship quality, physical appearance, level of playfulness, and inherent gender and/or sexual queerness. These features were displayed uniquely in the dynamic of the couple—a dynamic that interviewees wanted to be a part of. It is unknown how the fantasy to “participate” in the dynamics of the couple translates into a reality. It also unknown if this fantasy is purely motivated by a desire to be a in a sexual or romantic dynamics with couples or if it is also inspired by other factors such as a desire to have a relationship like the one they perceive the couple to have, a perceived lack of responsibility as the role of the third, a perceived heightened experience of validation from being included by a couple, or perhaps a form of voyeurism. Research is needed on the primary motivations of symbiosexual desire. Research is also needed on the nature and quality of people’s sexual and romantic experiences with couples and if these experiences uniquely or more affectively meet the needs of individuals with symbiosexual attractions. Further, research is needed on how these experiences may relate to and affirm the salience of symbiosexual attraction.

Interviewees also expressed hesitation and uncertainty about symbiosexual desire. Cameron questioned whether his attraction to couples was “psychologically healthy.” Explanations for interviewee uncertainty and concern was beyond the scope of this study. However, it is likely that lack of language for this attraction, its departure from mononormative assumptions of attraction to individuals, and the critiques leveled at those in who purse dynamics with couples in polyamory communities are contributing factors. Specifically, the stigma in the polyamory community that it is unhealthy and/or dangerous to pursue dynamics with established couples or unicorn hunters (Johnston, 2024) may be influencing people’s perceptions of those that desire sex and relationships couples (sometime labeled as unicorns). More research is needed to assess the validity of the ethical concerns and critiques associated with this desire, as well as how social stigma impacts people who experience symbiosexual attraction.

Further, research is needed on what characteristics and/or life experiences inspire symbiosexual attraction. Is this attraction associated with a history of unhealthy or abusive romantic relationships? Is it associated with a specific relationship structure, or lack thereof, between a person’s primary caregivers? Interviewees highlighted relationship health as one the elements they were drawn to between couples. They specifically talked about relationships that were caring, loving, attentive, and responsive. Some found these characteristics appealing because they were foreign to other relationships they had experienced and/or witnessed. It is also possible that some found them appealing because they were familiar positive experiences, either in previous relationships or perhaps even from childhood. Because of fondness or absence, it is possible that symbiosexual desire reflects a yearning for the socially idealized childhood experience of having a healthy container of two loving adults. More research is needed on this possibility and if engagement in sex and relationship dynamics with couples can serve unique therapeutic function (as implied by interviewees who express desire for “healthy” couples), as well as what mental/emotion health concerns, if any, are addressed or emerge in these dynamics.

Regardless of why people experience this attraction or if this attraction is always “healthy,” the experience of symbiosexual attraction is a valid lived experience requiring language and recognition. While this experience is not necessary indicative of a preference or orientation toward people in relationships, its existence affirms the argument made by Klesse (2014) and others that multipartner, multidirectional sexual preferences may be a component of one’s sexuality. Similar to Galupo’s (2018) findings within bisexual populations, symbiosexual recognition and visibility will validate people’s lived experiences with this attraction. Further, empowered through language and the validation that this experience is real and felt by diverse populations, people who experience this attraction will be more likely seek sexual and romantic fulfillment based on their desires and preferences. It their examination of identity work in polyamorous communities, Ritchie and Barker (2006) found that development of new or rewritten language for nonmononormative lived experiences with sex and relationships can not only affirm but “enable alternative ways of being” (p. 596).

### Symbiosexual Attraction Is an Unfamiliar and Unexpected but Potentially Significant Experience

Survey and interviewee responses support H3, which hypothesizes that some people have experiences of symbiosexual attraction that are strong, significant, and/or pervasive. This finding suggests that some people may find the term symbiosexual useful to describe not only an experience of attraction but a lived experience of sexuality or even to describe a sexual orientation. In their attention to multigender and fluid orientations (Ahmed, 2006; Diamond, 2008, 2016; Hayfield, 2021; van Anders, 2015), queer sexuality scholars have offered applicable frameworks for considering symbiosexuality as an orientation or as part of one’s sexual configuration. More research is needed on the significance of symbiosexual attraction. If some people experience it as a consistent or strong component of their sexuality, how might the term resonate for them? Further, while outside the scope of this study, several interviewees mentioned desire toward groups and orgies. Research on experiences of attraction to relationships between more than two people, for example an attraction to a triad or larger groups, is needed to investigate broader conceptions of symbiosexuality.

Interviewee responses also revealed that experiencing this attraction can be surprising or unexpected. This finding is unsurprising given the lack of discourse on this attraction. More research is needed on how people make sense of their experiences of attraction to people in relationships in the context of their sexual orientation and the sociocultural messages and information they have received about sexuality.

### Limitations

There were several limitations of this exploratory study of symbiosexual attraction. I analyzed qualitative and quantitative data collected from *The Pleasure Study* (Harvey et al., 2023), which used convenience and snowball sampling. Therefore, findings cannot be generalized to the broader population. Further, the sample size available from this study, 145 survey participants and 34 interview participants, was small. It should be noted, however, that for queer populations 30–50 participants has been found to be a productive sample size for research studies (Compton, 2018). In addition, the sample was biased by those who were specifically recruited by *The Pleasure Study* (queer and nonmonogamous populations), those who self-selected to participate in a survey and an interview about sex and sexuality, and those who chose not to answer specific questions. Any conclusions drawn from these data are tentative and preliminary, and at risk of incorrect assessment due to nongeneralizable data, the limited amount of data, and the biases mentioned above.

### Conclusion

This study provided evidence of symbiosexual attraction and offered rich descriptions of how people experience this attraction. Like Giuliani (2021), participants in this study described a desire toward a dynamic, something greater than the sum of a couple’s parts. Harmoniously, the design of this study, an integrated mixed-methods analysis, produces findings that “are greater than the sum of their parts” (Woolley, 2009, p. 23). The diversity of people who reported experiencing attraction to the dynamic between couples as well as the frequency and strength with which some reported this experience has implications both for those who experience symbiosexual desire and for those who interact with this population, including partners, family members, community members, therapists, clinicians, and researchers. The ongoing invalidation, stigma, and discrimination, particularly within the polyamory community, directed toward people who are interested in sex and relationships with couples must be examined and challenged. Recognition and validation of symbiosexuality will offer support for this sexual minority both in the communities specifically formed to support people with marginalized sexual and relationship orientations (such as the polyamorous community) and in mainstream community settings.

Evidence provided in this study also has implications for the conceptualization of sexual attraction and sexual orientation within sexuality studies. Queer sexuality scholars continue to challenge and expand erotic possibilities by introducing new terms and descriptions of human sexual desires and sexual orientations. Their work has made space for conceptions of multigender and fluid orientations (Ahmed, 2006; Diamond, 2008, 2016; Hayfield, 2021; van Anders, 2015) as well as orientations not defined by sexual attraction (Bogaert, 2004, 2015). Sexual desires and orientations in these studies are conceptualized as a single line of attraction between one being and another, whether that line is straight, slanted, or “wonky” (Ahmed, 2006, p. 66). Descriptions in this study of symbiosexual attraction, as orientations toward a field of energy or toward a dynamic that is multidirectional or multiobject, push the boundaries of the concept of desire beyond singles lines of attraction toward single objects. These descriptions offer new possibilities for the conceptualization of sexual desire and orientation and serve the queer feminist agenda to undo sexual hierarchies in favor of benign sexual variation (Rubin, 1984) in lived experiences of sexuality.

## Data Availability

Not applicable.

## Appendix A: Survey Questions

**Table:**

• Gender Identity Label I most commonly use (select all that apply)—Selected Choice • The sexual orientation label that best describes me is (select all that apply)—Selected Choice • Do you identify as monogamous, polyamorous, other (please specify)
• Have you ever felt sexually/romantically attracted to a couple (two people and their relationship together, not each of them individually)?
Regarding your attraction to couples (two people in a relationship together)…
• I have experienced this attraction……(once, a few times, sometimes, often)
• What is your age in years (e.g. 22)
• What is the highest level of school you have completed or the highest degree you have received?
• Choose one or more races / ethnicities that you consider yourself to be:—Selected Choice
• Which best describes your social class now • In which country do you live?
• In which state do you live?
• Do you currently live in a rural or urban area?
• Describe your current religion, spiritual practice, or existential worldview?
• Is religion important to you

*Note *Questions only include a small portion of the survey questions from Stage 2 of The. Pleasure Study (Harvey et al., 2023).

## Appendix B: Interview Questions

### Interview Guide

#### Section 2: Attractions

People attracted to couples You indicated on the survey that you experience attraction to couples, when you have experienced this attraction, what about the couple(s) was attractive to you?

Prompt: What was it about them as a COUPLE (their relationship together) that was attractive?

*Note *Questions only include a small portion of the interview questions (one question with. prompts from Sect. 2 of the interview guide) from Stage 2 of The Pleasure Study (Harvey et al., 2023).

## Footnotes

1 Compersive describes having feelings of compersion—a term for the range of positive emotions such as joy, arousal, and contentment that some people experience as a witness to intimate relations between others, with whom they also share intimate connections (Thouin-Savard & Flicker, 2023).
